# Effect of Multiple Structural Parameters on the Performance of a Micromixer with Baffles, Obstacles, and Gaps

**DOI:** 10.3390/mi14091750

**Published:** 2023-09-07

**Authors:** Jiacheng Nai, Feng Zhang, Peng Dong, Fan Bai, Ting Fu, Jiangbo Wang, Anle Ge

**Affiliations:** 1 Hubei Key Laboratory of Mechanical Transmission and Manufacturing Engineering, Wuhan University of Science and Technology, Wuhan 430081, China; naijiacheng@foxmail.com; 2 Key Laboratory of Metallurgical Equipment and Control Technology, Ministry of Education, Wuhan University of Science and Technology, Wuhan 430081, China; dongpeng2532@foxmail.com; 3 School of Science, Wuhan University of Science and Technology, Wuhan 430081, China; baifan@wust.edu.cn; 4 Precision Manufacturing Institute, Wuhan University of Science and Technology, Wuhan 430081, China; futing1234gh@wust.edu.cn (T.F.); qdgwbs@163.com (J.W.); 5 Single-Cell Center, Qingdao Institute of Bioenergy and Bioprocess Technology, Chinese Academy of Sciences, Qingdao 266101, China; geal@qibebt.ac.cn

**Keywords:** micromixer, baffle, obstacle, mixing index, numerical simulation

## Abstract

As an essential component of chip laboratories and microfluidic systems, micromixers are widely used in fields such as chemical and biological analysis. In this work, a square cavity micromixer with multiple structural parameters (baffles, obstacles, and gaps) has been proposed to further improve the mixing performance of micromixers. This study examines the comprehensive effects of various structural parameters on mixing performance. The impact of baffle length, obstacle length-to-width ratio, gap width, and obstacle shape on the mixing index and pressure drop were numerically studied at different Reynolds numbers (*Re*). The results show that the mixing index increases with baffle length and obstacle length-to-width ratio and decreases with gap width at *Re* = 0.1, 1, 10, 20, 40, and 60. The mixing index can reach more than 0.98 in the range of *Re* ≥ 20 when the baffle length is 150 μm, the obstacle length-to-width ratio is 600/100, and the gap width is 200 μm. The pressure drop of the microchannel is proportional to baffle length and obstacle length-to-width ratio. Combining baffles and obstacles can further improve the mixing performance of square cavity micromixers. A longer baffle length, larger obstacle length-to-width ratio, narrower gap width, and a more symmetrical structure are conducive to improving the mixing index. However, the impact of pressure drop must also be considered comprehensively. The research results provide references and new ideas for passive micromixer structural design.

## 1. Introduction

Microfluidic chip (also known as lab-on-chip) is a scientific technology that operates fluids on a micrometer scale [1,2,3,4]. They are mainly used for basic operations such as sample preparation, reaction, separation, detection, and cell culture in fields such as chemistry, biology, and medicine [2,3,4,5,6,7,8]. A micromixer is a microfluidic device that is used to achieve rapid mixing of samples in microfluidic chips. Based on the difference of input capabilities, micromixers are classified into active and passive micromixers [9,10,11,12,13,14]. Active micromixers have higher mixing efficiency, and the mixing of fluid is driven by external driving energy such as electric fields [15,16,17,18], magnetic fields [7,19,20], acoustic fields [21,22], and light sources [23]. Thus their manufacturing costs are high, as well as requiring more complex design and processing. Sasaki et al. [17] used a pair of coplanar electrodes with a sine-wave gap to facilitate mixing, and the effects of salt concentration and frequency of applied voltage on mixing efficiency were studied by conventional dilution experiments with using Texas Red-labeled dextran. Ryu et al. [20] reported a micromagnetic stirring rod mixer driven by an external rotating magnetic field; they verifies its rapid mixing performance and that the magnetic stirring rod can be used to pump liquid in microchannels. Zhang et al. [22] demonstrated a dexterous microfluidic device that can perform bio/chemical reactions on chip without volumetric restrictions by the integration of single layer valves (for reagent dispensing) and surface acoustic wave excitation (for rapid reagent mixing). Active micromixers have a simpler three-dimensional microstructure and faster mixing speed but also require higher processing costs and processes.

Passive micromixers can induce convection by optimizing and designing the microchannel structures, thereby breaking the laminar state of the fluid and increasing the contact area between fluids to achieve rapid mixing. The passive micromixer has the advantages of easy processing, easy integration, and low cost [24], ensuring it has become one of the research focuses for achieving efficient mixing in microfluidic chips. Current research on passive micromixers focuses on designing channel shapes, structures, and embedding obstacles, which produce stretching, folding, squeezing, and separation-recombination of fluid morphology, and enhancing mixing index to achieve efficient mixing through rational design of channel parameters. Setting baffles and obstacles in microchannels to produce chaotic convection is a common method for achieving mixing. Vahid et al. [25] studied the effect of different shapes and numbers of baffles on mixing in curved serpentine micromixers, finding that baffles can generate local small vortices in laminar flow, enhancing fluid disturbance, stretching, and folding, and thus improving mixing performance. Tohid et al. [26] proposed a new baffle structure based on topological optimization and studied the effect of the vortices generated by the new baffle on the convection mechanism. Xiong et al. [27] numerically simulated the effect of variable-angle grooves and baffles on the mixing efficiency of micromixers, promoting fluid mixing by designing grooves and baffles to interfere with fluid flow and prolong fluid residence time in the channel. Xia et al. [28] researched the comprehensive performance of micromixers based on the principles of multi-vortex, contraction-expansion, split-recombination, and using the synergy principle of velocity field and concentration field. Xie et al. [29] investigated the effect of staggered side-by-side and non-side-by-side baffles on fluid disturbance, promoting fluid mixing by generating a serrated fluid interface. In square-cavity passive micromixers, the design of baffles and obstacles is a widely researched scheme, with the influence of shape, size, and layout of obstacles and baffles being key research focuses on micromixer performance and flow characteristics. At present, most of the existing square-cavity passive micromixers are arranged with single or symmetrical structures for baffles and obstacles; however, there are few reports on the combined influence of multiple structural (baffles, obstacles, and gaps) on mixer simultaneously. The parameter influence rules for new micromixers still needs further exploration.

In this paper, a square cavity micromixer with rib structures is presented, in which multiple baffles and obstacles are arranged in the square cavity to form rib structures and connecting different square cavities through gaps. The squeezing effect of the narrow space produced by the baffles and obstacles on the fluid is combined with the throttling effect of the gaps, and thus generates chaotic convection between the two fluids in the mixing area, which increases the contact area between the fluids and achieves vortex enhancement. Visualization experiments and numerical simulations are used to study fluid flow and mixing characteristics. The effects of the parameters of baffles, the parameters and shapes of obstacles, and parameters of gaps on mixing performance are discussed through numerical calculations within the range of *Re* = 0.1, 1, 10, 20, 40, and 60. The optimal design parameters are obtained by considering both the mixing index and pressure drop.

## 2. Geometry of the Micromixer

**Figure 1:**
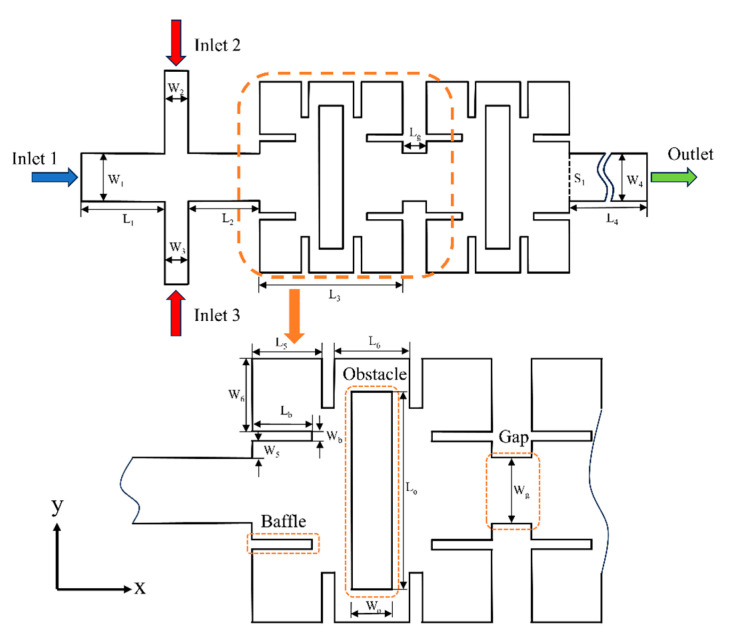
A two-dimensional sketch of the micromixer.

In this paper, a three-dimensional geometric model of a square cavity micromixer with baffles, obstacles, and gaps is established. The height of the micromixer is uniform throughout and fixed at 50 μm. The two-dimensional sketch is shown in Figure 1, which consists of a cross-shaped inlet, two rectangular square cavities with baffles and obstacles connected by gaps, and a rectangular outlet. Fluid A flows in through Inlet 1, fluid B through Inlet 2 and Inlet 3, and the mixed fluid flows exit through the Outlet. Cross-section S_1_ is defined as the outlet of the second rectangular square cavity; the mixing index of section S_1_ is taken as the research object in this research. The mixing area length of the micromixer is 1.13 mm, and the total length is 3.35 mm. Reducing the size of microchannels can effectively improve the mixing efficiency of molecular diffusion. It has been reported that in microchannels with a size of only a few microns, the mixing time of different components of the fluid is less than 100 ms, and the diffusion rate is fast. However, the presence of microchannels with excessively small dimensions can result in significant pressure drops and the possibility of channel blockages [30,31]. To ensure equal flow rates of fluid A and fluid B, the lengths of the inlet channels are all set as L_1_, and the width of Inlet 1 is W_1_ = 2W_2_ = 2W_3_. Table 1 lists the specific dimensions of the micromixer.

## 3. Numerical Model and Verification

### 3.1. Numerical Model

This study employs COMSOL Multiphysics 6.1 software to conduct three-dimensional numerical simulations of fluid flow and mixing properties. The physical field adopts laminar flow and transport of diluted species. To avoid numerical instability in convection-dominated transport, the default Consistent Stabilization is used for stability in the transport of diluted species. This method employs streamline diffusion and crosswind diffusion techniques. Additionally, to save computation, the equation residual is set to approximate residual. The fluid flow is presumed to be steady and laminar, and the fluid being treated as an incompressible Newtonian fluid. The influence of gravity is disregarded. Therefore, the governing equations for fluid flow within the micromixer adhere to the continuity equation and the Navier–Stokes equation, namely:

(1) $$\nabla \cdot \vec{v}=0,$$

(2) $$\rho \left(\vec{v}\cdot \nabla \right)\vec{v}=-\nabla p+\mu \nabla^{2}\vec{v},$$

where $\vec{v}$ is the velocity vector of the fluid, ρ is the density of the fluid, p is the pressure, and μ is the dynamic viscosity coefficient of the fluid. ρ is 998 kg/m^3^ and μ is 9.7 × 10^−4^ Pa·s in this model.

Regarding the solution of the velocity field, a no-slip wall is set on the wall of micromixer. The fully developed flow is set as the velocity boundary condition at the three inlets of the microchannel. The outlet is set the pressure boundary condition; the pressure is 0.

Assuming that no chemical reaction occurs between the two mixed fluids and ignoring the heat of dissolution between them, the mixing of solutes satisfies the convection-diffusion equation, which is:

(3) $$\frac{\partial C}{\partial t}+\vec{v}\cdot \nabla C=D\nabla^{2}C,$$

in this model, *C* represents the concentration of the solute to be mixed, and D represents the diffusion coefficient of the solute, *D* = 1 × 10^−11^ m^2^/s. The concentration boundary conditions are specified at the channel entrance, with $C_{1in}=1$ is set at the Inlet 1 boundary and $C_{2in}=0$ is set at the Inlet 2 and Inlet 3 boundaries. The concentration boundary condition at the outlet of the channel is as follows:

(4) $$\vec{n}\cdot (-D\;\nabla \;C)=0,$$

where $\vec{n}$ is the external normal unit vector.

The mixing index of S_1_ is used to evaluate the performance of the micromixer. The formula for calculating the mixing index is as follows [32]:

(5) $$\eta =\left[1-\frac{\iint_{S}\left|C-C_{\infty }\right|dydz}{\iint_{S}\left|C_{0}-C_{\infty }\right|dydz}\right]\times 100\%,$$

where C is the concentration distribution on the exit section, $C_{\infty }$ is the concentration at full mixing which is defined as 0.5, and $C_{0}$ = 0 is the concentration when the mixture is unmixed. The value of η is 0 < η < 100%, η = 0 indicates unmixed state, and η = 100% indicates fully mixed state.

The *Re* is also an essential parameter, defined as:

(6) $$Re=\frac{\rho vD_{h}}{\mu },$$

(7) $$D_{h}=\frac{4A}{P},$$

where v is the mean velocity of the fluid, $D_{h}$ represents the hydraulic diameter of the channel. A denotes the cross-section area of the channel, and P represents the perimeter of the channel section.

To ensure the accuracy of the numerical calculation results, a grid independence analysis is performed. The grid convergence index (GCI) [33] is used as the convergence criterion, with an absolute tolerance set to 0.001. The expression for GCI is as follows:

The grid convergence index (GCI) has been calculated to ensure the accuracy of the calculation results [33], and the absolute tolerance has been specified to be 0.001 as the convergence criterion. The expression of GCI is shown as follows:

(8) $$GCI=F_{s}\frac{\left|\varepsilon \right|}{r^{p}-1},$$

(9) $$\varepsilon =\frac{f_{coarse}-f_{fine}}{f_{fine}},$$

where $F_{s}$, p, and r are the safety factor, the order of accuracy of the numerical method, and grid refinement ratio, respectively. $f_{coarse}$ and $f_{fine}$ are the numerical results resulting from the coarse and fine meshes, respectively. $F_{s}$ takes the value of 3, as recommended in [33]. At *Re* = 10, the mixing index at the exit of the second square cavity (section S_1_) is used as the criterion to determine the Grid Convergence Index (GCI). The GCI is calculated for grid numbers of 26.9w, 77.6w, and 116.5w, with the results shown in Table 2. The GCI of the mixing index is found to be 0.7634. Based on this result, a grid number of 776,675 is chosen for the numerical solution.

### 3.2. Experimental Verification

**Figure 2:**
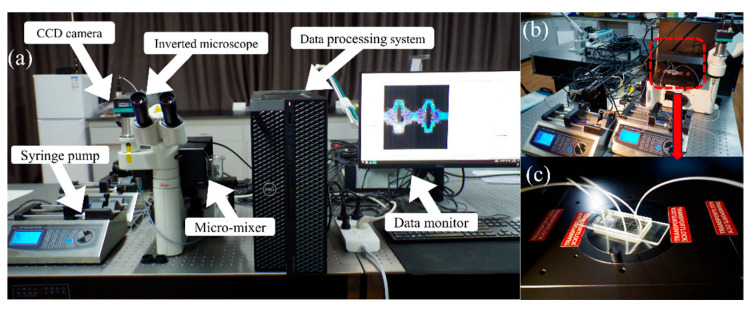
The photographs of Mirco-PIV. (**a**) Experimental system; (**b**,**c**) Microfluidic chip.

**Figure 3:**
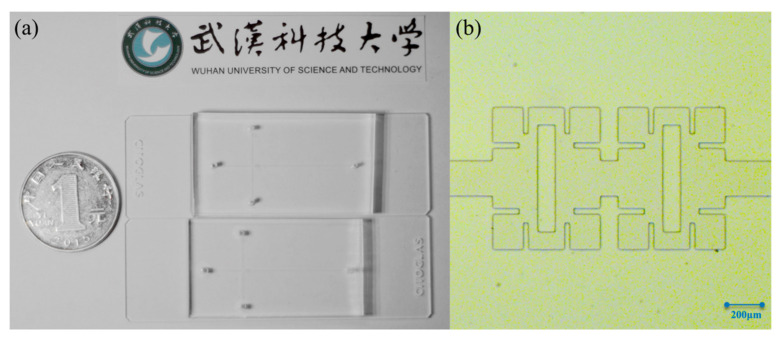
The photographs of the micromixer. (**a**) Photograph of the microfluidic chip; (**b**) Micrograph of the micromixer.

To verify the reliability of the numerical model, a microfluidic experimental platform and the Mirco-PIV system (Dantec Dynamics, Skovlunde, Denmark), shown in Figure 2, are used to conduct a visual experiment on the fluid flow state in the micromixer. The fluid flow characteristics in the micromixer are quantitatively analyzed and compared with numerical results. Figure 3 shows the microfluidic chip used in the experiment, made by bonding a polydimethylsiloxane (PDMS) etched with microchannels and a glass substrate (processed by the Qingdao Institute of Bioenergy and Process of the Chinese Academy of Sciences).

**Figure 4:**
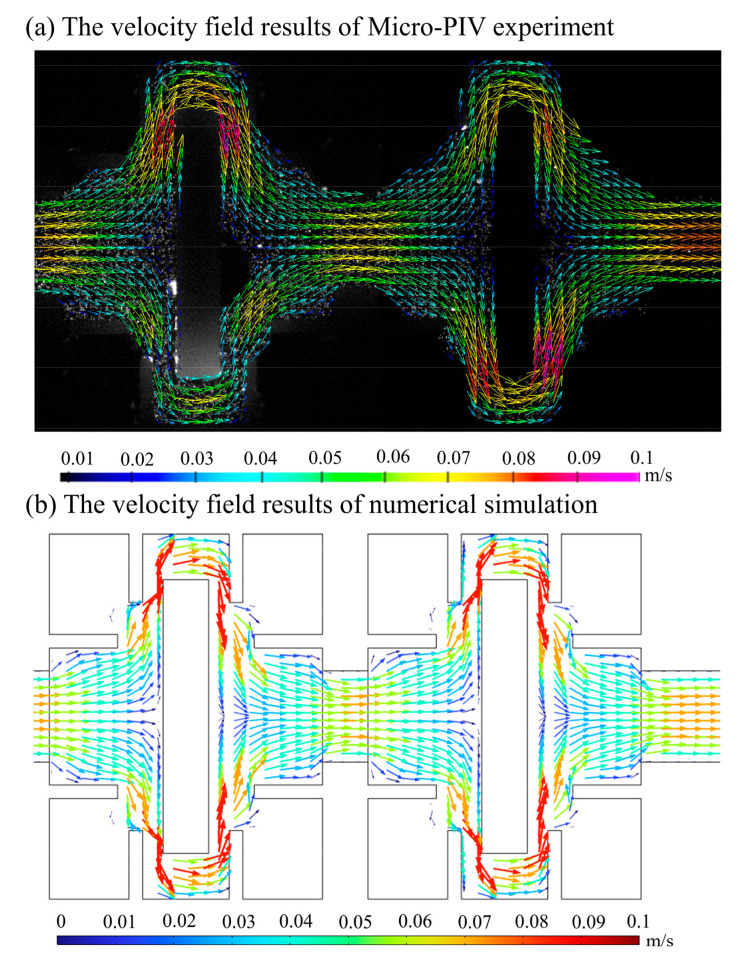
Fluid velocity field in micromixer. (**a**) The velocity field results of Micro-PIV experiment; (**b**) The velocity field results of numerical simulation.

Three micro-injection pumps (SP02-1B dual-channel extraction and perfusion type, Chuangdi Electronic Technology, Baoding, China) inject deionized water containing 1 μm tracer particles (FluoroSpheres, Thermo Fisher Scientific, Waltham, MA, USA) into the micromixer from three inlets. The fluorescent signal of tracer particles moving with the fluid in the channel is collected and processed by computer to obtain the fluid velocity vector field. Figure 3a,b show the physical map and optical microscope photograph of the micromixer, respectively. Figure 4a,b show the experimental results of fluid velocity field collected by Micro-PIV system and numerical results under the same conditions, respectively. The fluid flow patterns and streamlines shown by experimental and numerical results are well matched. Due to the very slow fluid flow at the corners of the mixer, the sample may remain and be difficult to replace. Such designs should be avoided in practical applications.

**Figure 5:**
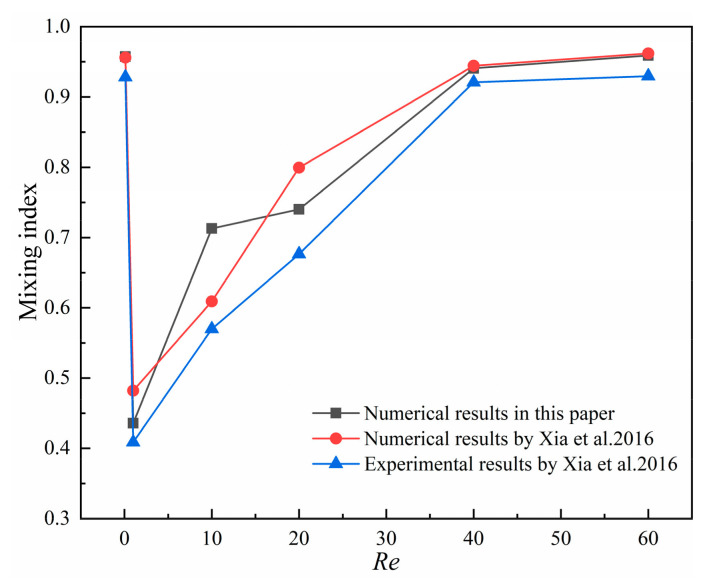
Comparison of numerical results of mixing index with results by Xia et al., 2016 [28].

Experimental results and numerical mixing index results from the square cavity micromixer in reference [28] are used to validate the computational model and numerical results presented in this paper. A 3D square cavity micromixer model with the same structure as in [28] is set up, and the comparison of numerical results of mixing index with results in [28] are presented in Figure 5. This figure shows the numerical mixing index results obtained by our computational model, as well as the experimental and numerical results from [28]. As shown in Figure 5, the numerical results are higher than the experimental data due to the assumptions in the simulation. At *Re* = 0.1, the very slow fluid flow rate makes the fluids of different components have a longer residence time in the channel, thus promoting sufficient mixing; the mixing indexes of the three curves are very good. In the range of *Re* = 1, 10, 20, 40, and 60, the mixing index gradually increases with *Re*, the mixing index results from our computational model correspond well with those in [28].

## 4. Results

**Figure 6:**
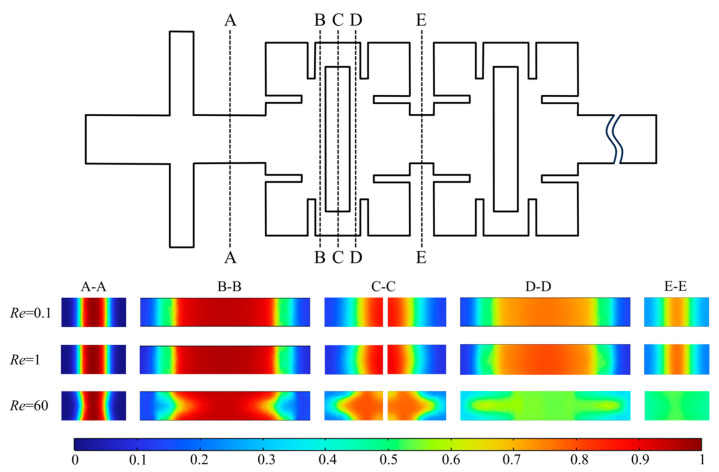
Concentration distribution at different y-z plans of micromixer for *Re* = 0.1, 1, and 60.

Figure 6 shows the concentration distribution in five y-z planes along the x direction at *Re* = 0.1, 1, and 60. Five cross-sections A-A, B-B, C-C, D-D, and E-E were selected to analyze the concentration field, and thus analyze the flow and mixing characteristics of two fluids in the micromixer.

The mixing effect is not obvious within the range of *Re* = 0.1 and 1 in cross-section A-A. The fluid initially contacts, and the interface between the fluids is obvious. At *Re* = 60, the contact surface between the two fluids is convex because of the different speed at the border and the center of the channel (the flow rate in the center of the channel is faster). In cross-sections B-B, C-C, D-D, and E-E, at *Re* = 60, the form of mixing changes from molecular diffusion to convection-dominated transport as the fluid velocity increases. The boundary between different component fluids becomes a curve due to the influence of the micromixer structure; fluid generates lateral movement by the action of baffles and obstacles. The fluid flows upward to the walls on both sides of the microchannel in the B-B section. After passing through the obstacles, the fluid flows to the center and confluence in the middle of the micro-channel in the D-D section. The boundary between different component fluids is blurred and curved. At *Re* = 60, the fluid contact surface is fuzzier and more tortuous than at *Re* = 0.1 and 1. The fluid is basically completely mixed when the fluid reaches the section E-E.

At *Re* = 0.1 and 1, the concentration distribution and obvious fluid boundaries in each section is basically consistent. The mixing of fluids mainly relies on molecular diffusion and is less affected by structure, resulting in poor mixing effect with obvious boundaries between different fluids. However, compared with *Re* = 1, the fluid velocity is slower and the residence time of the fluid in the channel is longer, resulting in longer mixing time and better mixing efficiency at *Re* = 0.1. At *Re* = 60, fluid boundaries in each section are curved and blurred; fluid mixing is mainly dominated by convective diffusion, with the fluid mixing thoroughly at the extrusion effect of the channel structure.

**Figure 7:**
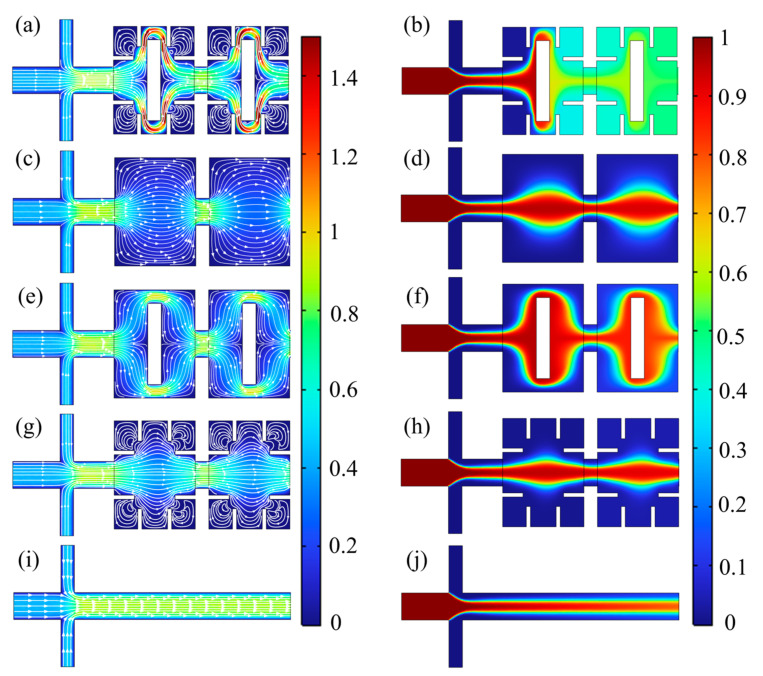
Velocity field distribution (m/s) and concentration field distribution of five micromixers at *Re* = 20. (**a**,**b**) New micromixer; (**c**,**d**) Square cavity micromixer without baffle or obstacle; (**e**,**f**) Square cavity micromixer with obstacles; (**g**,**h**) Square cavity micromixer with baffles; (**i**,**j**) Micromixer only with straight channel.

**Figure 8:**
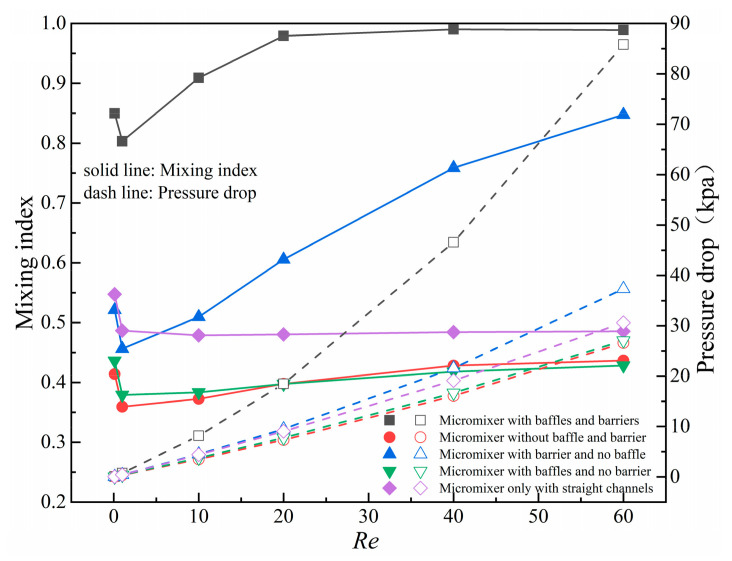
The mixing index and pressure drop curves of five micromixers at *Re* = 0.1, 1, 10, 20, 40 and 60.

To further study the mixing and pressure drop characteristics of the micromixer, a comparative analysis was conducted on a straight channel micromixer, a square cavity micromixer, a square cavity micromixer with baffles, a square cavity micromixer with obstacles, and the micromixer designed in this paper in order to investigate the combined effects of baffle structure, obstacles, and gaps. Taking *Re* = 20 as an example, Figure 7 shows the velocity field and concentration field distribution of five micromixers in the inlet and square cavity areas. Figure 8 presents the mixing index and pressure drop curves of five micromixers at *Re* = 0.1, 1, 10, 20, 40, and 60.

As shown in Figure 7a,b, with the new micromixer, the fluids are separated under the obstruction of obstacles after different components of fluid enter the rectangular cavity. The fluid generates lateral movement, and the lateral and longitudinal contact surface are elongated. The fluid is initially mixed under the squeezing action between the obstacles and baffles in the narrow space. When flowing through the obstacle area, the narrow space formed by the obstacle and wall reduces the cross-sectional area of microchannel, squeezing the fluid to promote mixing again. After flowing through the obstacle area, the fluid forms a jet under the squeezing of baffles and obstacles, merging in the middle of the square cavity. The mixing index can reach 0.98 at *Re* = 20 after two units of square cavity.

As presented in Figure 7c–h, the mixing efficiency is poor without the lateral movement caused by obstacles and squeezing effect of narrow gaps formed by obstacles and baffles. Fluid flows in layers with slower overall flow velocity, relying mainly on molecular diffusion for mixing. As can be seen in Figure 7c,d, the fluid expands and diffuses in rectangular cavities then contracts at gaps in the micromixer without baffles and obstacles. The mixing effect is worse without the acceleration and squeezing effects by narrow spaces or separation and the recombination effect by obstacles, with a mixing index only reaching 40%. The concentration distribution is similar to that of square cavity micromixers with only baffles, shown in Figure 7g,h, and thus their mixing index and pressure drop curves basically coincide. As indicated in Figure 7e,f, in a square cavity micromixer with only obstacles, fluid generates lateral movement after being separated by obstacles, and the contact area is elongated. At *Re* = 20, the mixing index can only reach 60%. In the micromixer only with straight channel shown in Figure 7i,j, the fluid flows in a layered state.

As presented in Figure 8, at *Re* = 0.1 and 1, the mixing index of the five micromixers shows a downward trend with *Re*. Fluid mixing is mainly dominated by molecular diffusion, which is less affected by structure. As *Re* changes from 0.1 to 1, the fluid velocity increases, resulting in shorter residence time of the fluid in the channel and shorter mixing time. Compared with ordinary square cavity micromixers (with only baffles, only obstacles, or no baffles and obstacles), the mixing index of the new micromixer has increased by 0.30~0.42. At *Re* = 10, 20, 40, and 60, the mixing index shows an upward trend with *Re*, the influence of natural diffusion on mixing efficiency gradually decreases, and chaotic convection dominated by structural influence plays a dominant role. Compared with micromixers with only baffles or only obstacles, the mixing index of the new micromixer has increased by 0.14~0.56. The pressure drop curves all show an approximately linear growth trend with the increase of *Re* and are positively correlated with mixing index.

## 5. Discussion

### 5.1. Effect of Baffle Length on Mixing Performance

**Figure 9:**
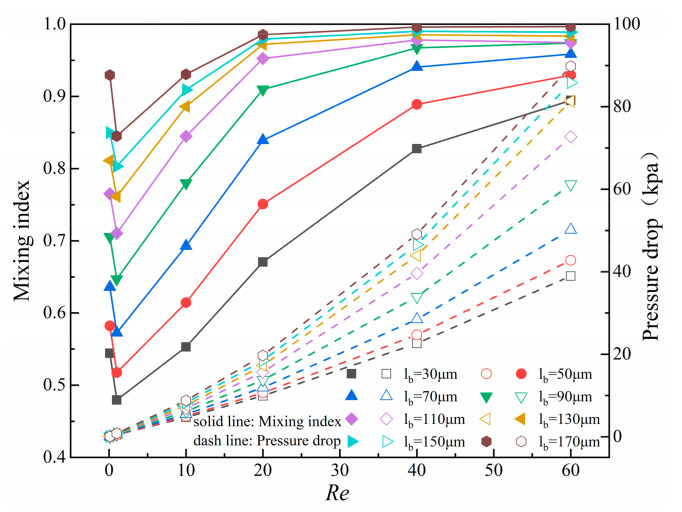
Curves of mixing index and pressure drop of micromixer under different baffle length and *Re*.

To explore the influence rule of baffle length on mixing index and pressure drop, eight values were selected within the range of 30~170 μm for baffle length. Figure 9 shows the curves of mixing index and pressure drop under different l_b_ at *Re* = 0.1, 1, 10, 20, 40, and 60. The mixing index shows a trend of decreasing and then increasing with *Re*, which is due to the transition from molecular diffusion to chaotic convection. The mixing index and pressure drop increase significantly with the gradual increase of l_b_. When l_b_ ≥ 150 μm, within the range of *Re* = 0.1 and *Re* ≥ 10, the mixing index reaches over 90%. At *Re* ≥ 20, the mixing index reaches over 95%, with good mixing efficiency in a wide range of *Re*. Compared with l_b_ = 150 μm, when l_b_ = 170 μm, the maximum increase in mixing index is 0.007, while the pressure drop increases by 3.99 kpa. After comprehensive consideration, l_b_ = 150 μm is selected as the design parameter.

As *Re* increases, fluid velocity gradually increases and obstruction effect on fluid by baffles and obstacles enhances, and thus the pressure drop also increases exponentially with *Re*. At the same time, the space formed between baffles and obstacles decreases with the increase of baffle length, which is also resulting in greater pressure drop.

**Figure 10:**
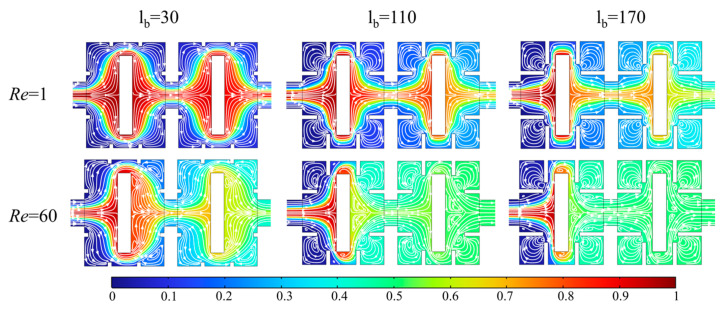
Concentration distribution and velocity streamlines of the fluid in the x-y plane when l_b_ = 30, 110, and 170 μm at *Re* = 1 and 60.

Taking *Re* = 1 and 60 as an example, Figure 10 shows the concentration distribution and velocity streamlines of the fluid in the x-y plane when l_b_ = 30, 110, and 170 μm. At *Re* = 1, the fluid flow velocity is slow, and thus there is no vortex generation in the channel. At *Re* = 60, fluid flow is significantly affected by structure; fluid generates backflow on both sides of the narrow space and expansion vortices between baffles due to the jetting effect of the narrow space between baffles and obstacles. However, the expansion vortex is close to the corner of the channel, and thus has a poor promotion effect on mixing.

When *Re* = 60, at l_b_ = 30 μm, only a small amount of backflow occurs behind obstacles with general mixing effect; the jetting effect of narrow spaces is enhanced with increases of l_b_. At l_b_ = 110 μm, the vortex generated by backflow is more obvious and a vortex is gradually formed in the space area between obstacles and walls with obvious mixing effect. It can be seen that this eddy current has obvious promoting effect on fluid mixing by comparing with *Re* = 1 (no eddy in the space area). At l_b_ = 170 μm, new vortices are generated on right side baffles of square cavity, fluid is basically completely mixed when flowing out of the first rectangular cavity. The obstruction effect of baffles and obstacles and throttling effect of formed narrow spaces can significantly form vortices in micromixer to efficiently promote fluid mixing by breaking fluid laminar state and boundaries.

### 5.2. Effect of Obstacle Aspect Ratio on Mixing Performance

To study the influence of obstacle structural parameters on mixing performance, the total area of obstacles is defined as 0.6 × 10^−8^ m^2^. Length-to-width ratios of L_o_/W_o_ = 750/80, 600/100, 500/120, 400/150, 375/160, and 300/200 were selected to discuss the influence of different obstacle width-to-length ratios on mixing performance.

**Figure 11:**
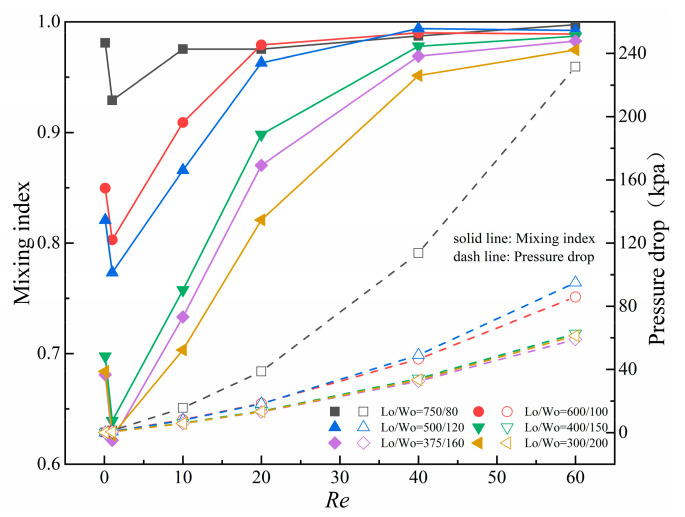
Curves of mixing index and pressure drop at different L_o_/W_o_ and *Re*.

Figure 11 shows the mixing index and pressure drop curves at different L_o_/W_o_ and *Re*. At the same L_o_/W_o_, the mixing index shows a trend of first decreasing and then increasing with *Re*, and pressure drop shows an increasing trend. In micromixers with different L_o_/W_o_, as *Re* increases, the maximum mixing index gradually moves toward a larger pressure drop (higher L_o_/W_o_). At *Re* ≥ 20, when L_o_/W_o_ = 600/100 and 500/120, the mixing index can reach over 90%. At L_o_/W_o_ = 750/80, mixing efficiency can reach over 90%, with a mixing index of 99.7% at *Re* = 60. However, compared with L_o_/W_o_ = 600/100 and 500/120, pressure drop increases by 79.1~169.9%, reaching to 231.6 kpa at *Re* = 60. Compared with L_o_/W_o_ = 500/120, L_o_/W_o_ = 600/100 has better mixing index and smaller pressure drop. Considering comprehensively the adverse effects of excessive pressure drop on pipelines, this paper selects obstacle length-to-width ratio L_o_/W_o_ = 600/100 for design.

**Figure 12:**
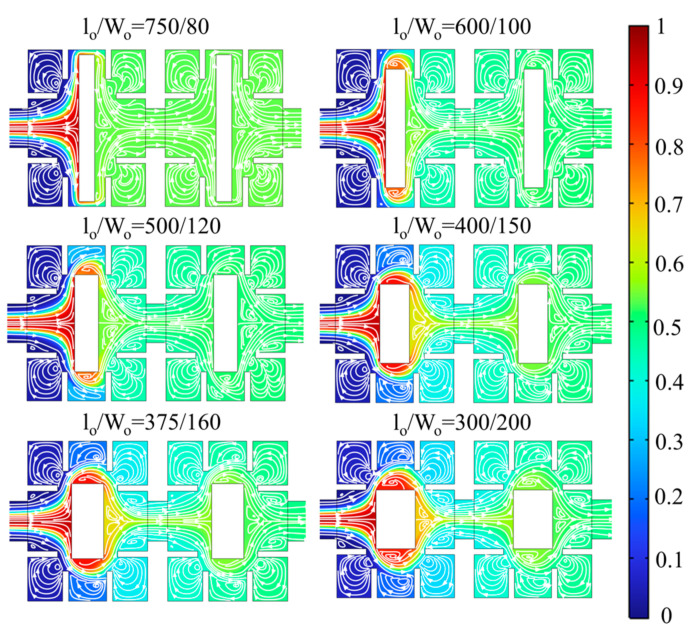
Concentration distribution and velocity vector of S_4_ cross section at *Re* = 60.

Figure 12 presents the concentration distribution and velocity streamlines in the square cavity area at different length-to-width ratios at *Re* = 60. When L_o_/W_o_ = 750/80, the space between obstacles and walls is only 50 μm, resulting in a small cross-sectional area of fluid flow and strong squeezing effect on fluid but also resulting a large pressure drop. When L_o_/W_o_ = 600/100 and 500/120, the overall concentration distribution and velocity streamline distribution in square cavities are consistent with similar mixing performance. The expansion vortices gradually form and grow between baffles on the side walls close to the wall as the length-to-width ratio gradually increases. However, these vortices have no significant effect on promoting mixing because they are too close to the wall. The squeezing effect on fluid weakens and mixing performance gradually decreases with the spaces between baffles and obstacles gradually increases. Therefore, compared with L_o_/W_o_ = 500/120, micromixers with L_o_/W_o_ = 600/100 have better mixing performance.

### 5.3. Effect of Gap Width on Mixing Performance

The micromixers have been able to achieve good mixing performance within a wide range of *Re* values under specific baffle lengths and obstacle length-width ratio. There is no significant improvement on the mixing index by continuing to change structural parameters. To discuss the impact of different gap widths on mixing performance, the effect of gap width is examined in the range of 50–200 μm under the condition of baffle length of 70 μm.

**Figure 13:**
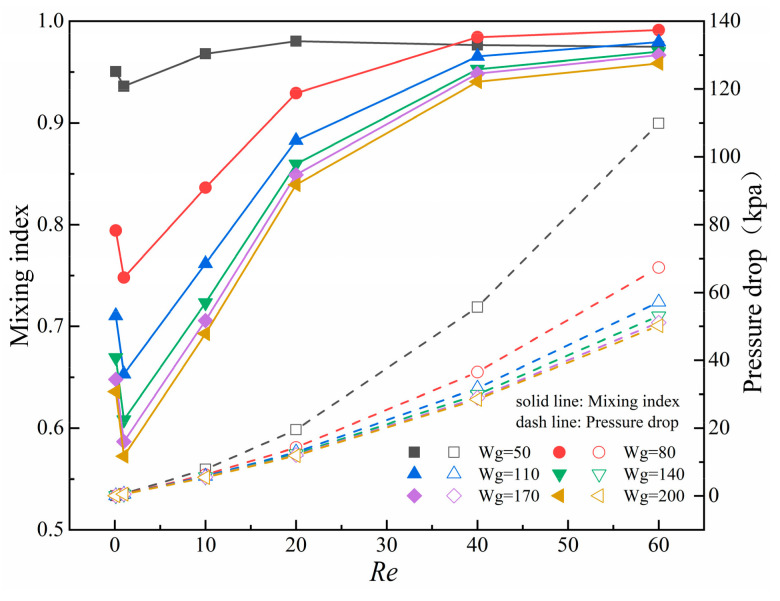
Curves of the mixing index and pressure drop with *Re* at different gap widths.

Figure 13 shows the curves of the mixing index and pressure drop with *Re* at different gap widths. It can be found that the mixing index increases as the gap width decreases. The overall mixing efficiency is best at W_g_ = 50 μm, reaching more than 95% in the range of *Re* = 0.1, 10, 20, 40, and 60. However, the pressure drop has increased by 1.29–2.19 times compared to when the gap width is wider (W_g_ = 200 μm). The transition point from molecular diffusion to chaotic convection is advanced under the decrease of W_g_.

**Figure 14:**
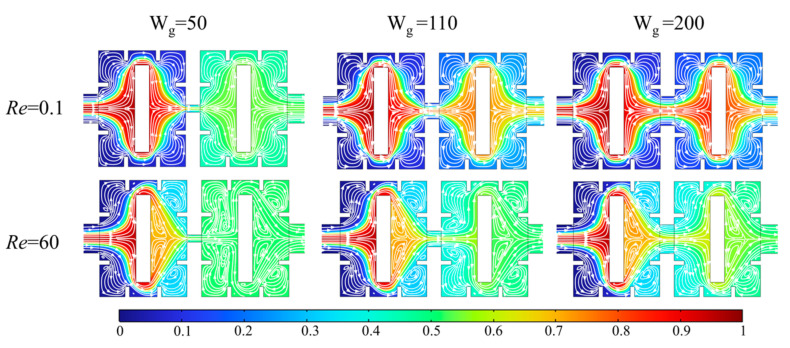
Effect of the number of baffles on mixing index at different *Re*.

When the gap width (W_g_) is 50, 110, and 200 μm, Figure 14 shows the concentration distribution and velocity streamline of the x-y cross-section of the micromixers at *Re* = 0.1 and 60. At *Re* = 0.1, there are no vortices in micromixers under different gaps, but the difference in mixing effects under different gap widths is obvious. At *Re* = 60, due to the increase in flow rate, the fluid impacts the obstacles and generates a backflow vortex at the gap outlet, and the size of the vortex gradually increases with the decreases of the gap width. The throttling effect of the gap is strongest at W_g_ = 50 μm, and the fluid is basically completely mixed at the gap area. However, a smaller gap width also produces a larger pressure drop. The pressure drop generated by the fluid is 2.19 times that of W_g_ = 200 μm, which causes a greater burden on the micromixer.

### 5.4. Effect of Asymmetric Obstacles on Mixing Performance

**Figure 15:**
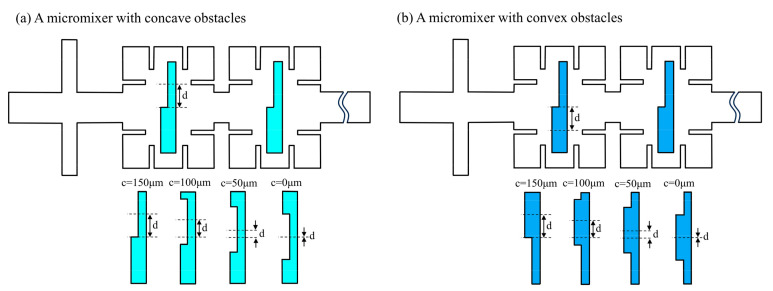
Micromixers with asymmetric obstacles. (**a**) A micromixer with concave obstacles; (**b**) A micromixer with convex obstacles.

As presented in Figure 15, the asymmetric micromixers with concave obstacles and convex obstacles with a groove of 300 μm × 50 μm was designed. The distance d between the centerline of the groove (or the convexity) and the centerline of the obstacle is used to research the influence of the structural parameters of the asymmetric obstacle on fluid flow and mixing characteristics. At d = 150 μm, the shape of the concave obstacle is in consistent with that of the convex obstacle, and the concave obstacles and convex obstacles are symmetric at d = 0 μm.

**Figure 16:**
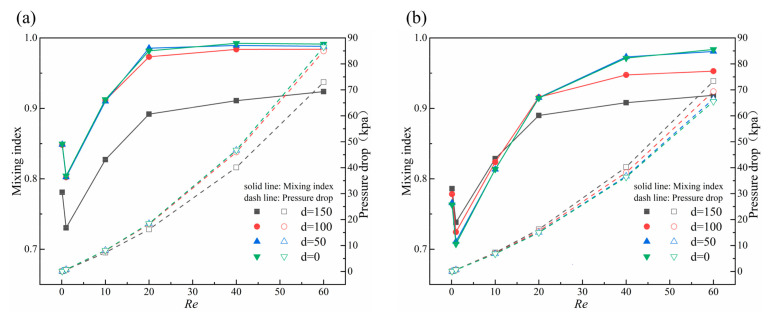
Curves of the pressure drop and mixing index of the micromixer with *Re* at different distances d. (**a**) Micromixers with concave obstacles; (**b**) Micromixers with convex obstacles.

Figure 16 shows the curves of the pressure drop and mixing index of the micromixer with *Re* at different distances d. Figure 16a shows the results of micromixers with concave obstacles. It can be seen that the mixing index and pressure drop are similar at d = 0, 50, and 100 μm, while d = 150 μm has the smallest mixing index and pressure drop, resulting in the worst mixing performance. Figure 16b shows the results of micromixers with convex obstacles. The mixing index increases as the asymmetry of the obstacle increases (the distance d increases) at low *Re* (*Re* = 0.1, 1, and 10), with d = 150 μm having the best mixing efficiency. As *Re* increases (*Re* = 20, 40 and 60), the mixing index increases as the symmetry of the obstacle increases (the distance d decreases), and d = 0 μm has the best mixing performance and the smallest pressure drop.

**Figure 17:**
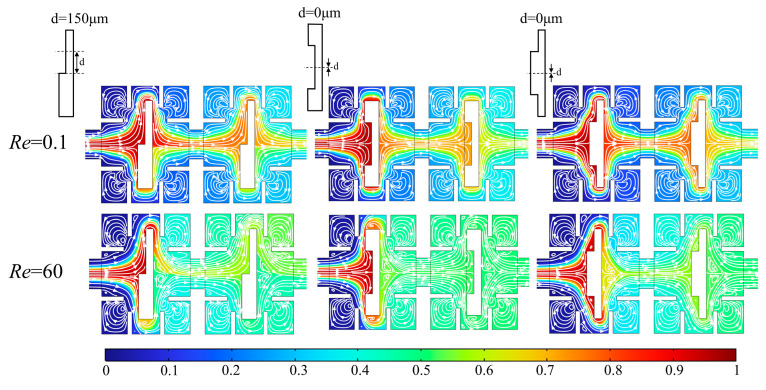
Concentration distribution and velocity streamline diagram of the micromixers with concave and convex groove obstacles when d = 0 and 150 μm at *Re* = 0.1 and 60.

Figure 17 shows the concentration distribution and velocity streamline diagram of the micromixers with concave obstacles and convex obstacles when d = 0 and 150 μm at *Re* = 0.1 and 60. At d = 150 μm, the shape of the concave obstacles is the same as the convex obstacles, and both the concentration distribution and velocity streamline are consistent. In the micromixers with concave obstacles the mixing effect in the upper part of the micromixer (the concave part of the obstacle) is worse at d = 150 μm compared to d = 0. The reason is that the space narrows between the baffles and the obstacle at the concave part becomes larger, thus weakening the squeezing effect on the fluid and reducing vortex generation on the upper side wall of the obstacle. When d = 0, obvious vortices are generated in both upper and lower wall areas of the obstacle, resulting in a blurred fluid interface and obvious mixing effects.

In the micromixers with convex obstacles at *Re* = 0.1, d = 150 μm and 0, there are generally consistent mixing trends in the upper half part of micromixers due to the larger space narrow between the concave groove of the obstacles and baffle. When d = 150 μm, there is a significant improvement in mixing effect due to well squeezing effect of obstacles squeezing baffles in lower half area, although the difference is not much compared with that when d = 0, where the overall mixing performance is better. At *Re* = 60, fluid flow is large and basically completely mixed after passing through the first rectangular cavity. Under asymmetric obstacles (d = 150 μm), the mixing in lower half part of the microchannel is further enhanced, and the mixing in upper half part of the microchannel is further weakened because of the asymmetrical eddy currents on the back side of the obstacle. Therefore, the overall mixing index is worse compared to symmetric obstacles (d = 0). As such, symmetric obstacles are more conducive to overall mixing performance in the micromixers designed in this paper.

## 6. Conclusions

In this paper, a new square cavity micromixer that incorporates baffles, obstacles, and gaps is proposed to examine the impact of various structural parameters on mixing performance through numerical analysis of the flow field, concentration field, pressure drop, and mixing index within the micromixer. Based on experimentally verifying the accuracy of the numerical model, the combined effects of baffle parameters, gap parameters, obstacle parameters, and shape on the micromixer were discussed.

Numerical results indicate that within the range of *Re* = 0.1 and 1, molecular diffusion is the primary mixing mechanism, and the mixing index is related to the residence time of fluid in the channel. At *Re* = 10, 20, 40, and 60, fluid convection becomes dominant, and the channel structure is the main factor affecting mixing performance.

A comprehensive study of multiple structural parameters reveals that the narrow space generated by the combination of baffle and obstacle will squeeze and jet the fluid, which forms a series of vortices and backflow areas on the wall and inside the micromixer. This effectively reduces the cross-sectional area of fluid flow and increasing the lateral contact area, and thus significantly strengthens mixing. The mixing index can exceed 97.9% in a wide range of *Re* (*Re* ≥ 20). Although increasing the length of the baffle and length-to-width ratio of obstacles or reducing gap width can improve the mixing index further, it also produces excessive pressure drop that affects fluid flow characteristics. Excessive pressure drop is not conducive to design and sealing of microchannel. Designing an asymmetric obstacle structure to generate asymmetric vortices in the channel can enhance mixing in half of the area but reduce it in the other half area, thereby reducing overall mixing performance.

In this paper, the effects of multiple structural parameters (baffles, obstacles, and gaps) on mixing performance are studied, and a micromixer is designed based on this analysis and comprehensively analyzed for mixing index and pressure drop. This provides a clear reference for passive micromixer structural design.

## Figures and Tables

**Table 1:** Structural parameters of rhombic separation and recombination micromixer.

Structure	Size (μm)
Cross-shaped inlet width (*W*_1_, *W*_2_, *W*_3_)	200, 100, 100
Inlet channel length (*L*_1_)	350
Length of the square cavity from the inlet (*L*_2_)	100
Square cavity length (*L*_3_)	600
Gap length (*L_g_*) Gap width (*W_g_*)	100 200
Mixing main outlet length (*L*_4_)	1300
Mixed main outlet width (*W*_4_)	200
Baffle length (*L_b_*)	150
Baffle width (*W_b_*)	30
Obstacle length (*L_o_*)	600
Obstacle width (*W_o_*)	100
Width of the baffle from the main channel (*W*_5_)	50
Width of the transverse baffle from the boundary of the square cavity (*W*_6_)	220
Length of radial baffle from the boundary of the square cavity (*L*_5_)	175
Transverse baffle spacing length (*L*_6_)	190

**Table 2:** The calculated data of grid convergence index.

The Number of Grid Control Units (N_1_/N_2_/N_3_)			Mesh Refinement Rate (r)	Relative Error (ε)	Safety Factor (F_S_) [33]	The Order of Accuracy (p)	Grid Convergence Index (GCI)
269,491	776,675	1,165,888	2.5	−0.026	3	1.608	0.7634

## Data Availability

Data sharing not applicable.
